# Preparation and Characterization of Biocompatible Iron/Zirconium/Polydopamine/Carboxymethyl Chitosan Hydrogel with Fenton Catalytic Properties and Photothermal Efficacy

**DOI:** 10.3390/gels9060452

**Published:** 2023-05-31

**Authors:** Xiaoyi Zheng, Hang Wu, Shige Wang, Jiulong Zhao, Lianghao Hu

**Affiliations:** 1 Postgraduate Training Base in Shanghai Gongli Hospital, Ningxia Medical University, Pudong New Area, No. 219 Miao Pu Road, Shanghai 200135, China; 2 Department of Gastroenterology, Changhai Hospital, Naval Military Medical University, No. 168 Changhai Road, Shanghai 200433, China; 3 School of Materials and Chemistry, University of Shanghai for Science and Technology, No. 516 Jungong Road, Shanghai 200093, China; sgwang@usst.edu.cn

**Keywords:** hydrogel, photothermal therapy, chemodynamic therapy, Fenton reaction

## Abstract

In recent years, multifunctional hydrogel nanoplatforms for the synergistic treatment of tumors have received a great deal of attention. Here, we prepared an iron/zirconium/polydopamine/carboxymethyl chitosan hydrogel with Fenton and photothermal effects, promising for future use in the field of synergistic therapy and prevention of tumor recurrence. The iron (Fe)–zirconium (Zr)@ polydopamine (PDA) nanoparticles were synthesized by a simple one-pot hydrothermal method using iron (III) chloride hexahydrate (FeCl_3_•6H_2_O), zirconium tetrachloride (ZrCl_4_), and dopamine, followed by activation of the carboxyl group of carboxymethyl chitosan (CMCS) using 1-(3-Dimethylaminopropyl)-3-ethylcarbodiimide hydrochloride (EDC)/N(4)-hydroxycytidine (NHS). Finally, the Fe–Zr@PDA nanoparticles and the activated CMCS were mixed to form a hydrogel. On the one side, Fe ions can use hydrogen peroxide (H_2_O_2_) which is rich in the tumor microenvironment (TME) to produce toxic hydroxyl radicals (•OH) and kill tumor cells, and Zr can also enhance the Fenton effect; on the other side, the excellent photothermal conversion efficiency of the incorporated PDA is used to kill tumor cells under the irradiation of near-infrared light. The ability of Fe–Zr@PDA@CMCS hydrogel to produce •OH and the ability of photothermal conversion were verified in vitro, and swelling and degradation experiments confirmed the effective release and good degradation of this hydrogel in an acidic environment. The multifunctional hydrogel is biologically safe at both cellular and animal levels. Therefore, this hydrogel has a wide range of applications in the synergistic treatment of tumors and the prevention of recurrence.

## 1. Introduction

The effective treatment of tumors remains a challenge for the biomedical field today [1]. Traditional clinical treatments include surgery [2], chemotherapy [3], radiotherapy [4,5], immunotherapy [6,7], etc. However, all these methods have certain limitations such as the risk of metastasis or infection due to the surgical trauma [8], the lack of targeting of some chemotherapeutic drugs to the lesion, and the killing of normal tissue cells [9,10]. These shortcomings have limited the prospects for their use in oncology treatment. Therefore, there is a pressing want to develop more effective therapeutic strategies to tackle the difficulties in tumor therapy.

In recent years, studies have proven that various kinds of tumor cells show off multiplied ranges of reactive oxygen species (ROS) and altered redox status due to genetic, metabolic, and microenvironmental alterations [11,12,13,14]. Stimulated by high ROS levels, oncogenes can induce the activation of various downstream signaling pathways to adapt to the oxidative stress environment [15,16,17]. This further leads to cell immortalization and contributes to the necessary conditions for the cells to reach a malignant growth state [18,19]. As a result, ROS-based antitumor strategies have gained widespread attention. Chemodynamic therapies (CDT) is a novel strategy for cancer treatment that involves multiple transition metal ions, such as iron (Fe), silver (Ag), molybdenum (Mo), ruthenium (Ru), cerium (Ce), and zinc (Zn) [20,21]. These metal ions can react with the endogenous hydrogen peroxide (H_2_O_2_) in the tumor microenvironment (TME) and produce a large number of hydroxyl radicals (•OH) to kill tumor cells [22,23,24,25]. Inspired by this promising therapeutic paradigm, iron-based nanoplatforms have been frequently studied for the treatment of tumors. Specifically, due to the anaerobic conditions at the tumor site, anaerobic enzymolysis and proton pumping at the cell membrane result in an acidic environment in the TME, and some TMEs exhibit high H_2_O_2_ expression [26]. CDT has several advantages over other therapies in that it is tumor-targeted, has few side effects on normal tissues, and can selectively kill tumor cells without relying on exogenous stimuli [23].

Photothermal therapy (PTT) has promising applications in the treatment of tumors. PTT refers to the use of photothermal agents with photothermal conversion efficiency and converts the near-infrared laser into heat [27,28,29]. This warmness can enlarge tumor temperature, cause irreversible injury to tumor cell membranes, and set off protein denaturation, leading to irreversible cell harm and subsequent tumor regression [30,31]. Currently, the main conjugated polymers (CPs) available for tumor PTT include polydopamine (PDA), polypyrrole, and polyaniline (PAI) [32,33,34,35]. Among them, PDA is a novel polymer assembled from dopamine monomers through oxidative autopolymerization [36,37]. It has been broadly used in tumor-targeted drug delivery systems due to its advantages such as good biocompatibility, excellent photothermal conversion properties, and multiple drug release response possibilities [36,38,39].

Hydrogels are three-dimensional porous structures produced from hydrophilic polymers by physical or chemical cross-linking methods [40]. Hydrogels can effectively encapsulate molecules and control their concentration at the lesion site [41]. In addition, hydrogels can respond to physical, biological, chemical, or external stimuli [42,43]. In this study, Fe ions are used as the classical Fenton agents [44,45], which can react with H_2_O_2_ at the tumor site to produce highly reactive •OH for tumor cells killing. Moreover, Zr ions that can synergize with Fe ions were introduced [46]. As a ligand for metal ions, PDA was employed to form noncovalent bonds with the nanoparticle surfaces and modulate the photothermal properties [47]. Then, the EDC/NHS activated carboxymethyl chitosan (CMCS) is used to combine with Fe–Zr@PDA nanoparticles to form a drug reservoir. Together with the CMCS hydrogel drug reservoir, this nanoplatform can be locally implanted to the target site to react with the H_2_O_2_, transform the laser to heat, and release the drug. Specifically, we introduced PDA with Fe–zirconium (Zr) to impart unique photothermal properties to the nanomedicine. In brief, Fe–Zr@PDA nanoparticles were mixed with carboxymethyl chitosan (CMCS) activated by 1-(3-Dimethylaminopropyl)-3-ethyl carbodiimide (EDC)/N-hydroxysuccinimide (NHS) to form a hydrogel.

**Scheme 1:**
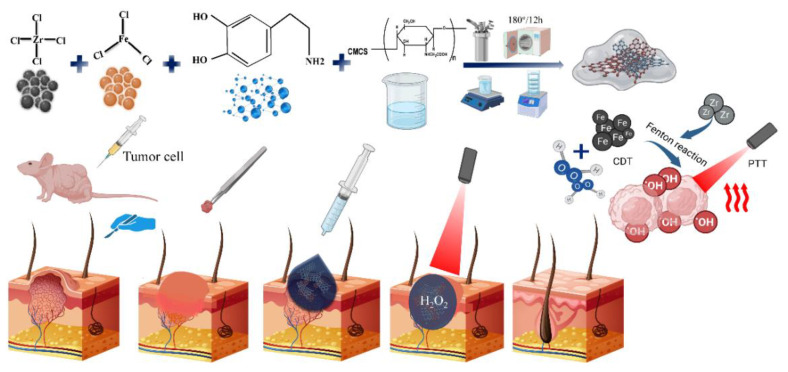
A practical and working schematic of a multifunctional hydrogel that can be injected for future use in killing tumors and inhibiting their postoperative recurrence (CDT: chemodynamic therapies; PTT: photothermal therapy).

In this work, an Fe-ions-mediated Fenton reaction was combined with the synergistic effect of Zr ions and the photothermal effect of PDA. Together with the CMCS hydrogel drug reservoir, this nanoplatform can be locally implanted to the target site to react with the H_2_O_2_, transform the laser to heat, and release the drug. Therefore, the composite hydrogel is expected to improve the efficacy of oncology treatments via preventing the postoperative tumor recurrence and increase patient compliance in future potential clinical applications. In summary, our aim is to combine the classical Fenton effect of Fe-based nanomaterials, the synergistic effect of Zr, the photothermal properties of PDA, and the bicompatible advantages of hydrogels to form a composite hydrogel. The composite system provides a new idea in broadening the application potential of hydrogels. While combining the Fenton effect and photothermal efficacy, the functionalized hydrogel can not only function as a tumor suppressor, but also plays an important role in preventing the recurrence of residual tumor tissue after local surgical excision (Scheme 1). The novelty of our work lies in the use of the classical Fenton reaction, combined with metal ion properties, to complement the hydrogel to form a composite multifunctional hydrogel, circumventing the disadvantages of applying nanoparticles alone and amplifying their advantages to provide a novel strategy in the field of tumor killing and prevention of local tumor recurrence.

## 2. Results and Discussion

### 2.1. Preparation and Characterization of Fe–Zr@PDA and Fe–Zr@PDA@CMCS Hydrogel

**Figure 1:**
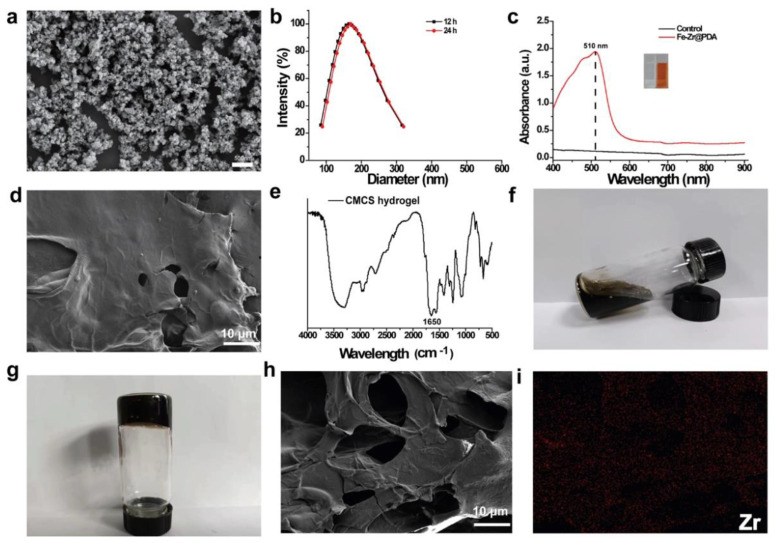
(**a**) SEM image of Fe–Zr@PDA nanoparticle; (**b**) the hydration kinetic diameter of Fe–Zr@PDA in H_2_O; (**c**) the light absorption of phenanthroline solution with and without co-incubation with Fe–Zr@PDA; (**d**) SEM image of CMCS hydrogel; (**e**) FTIR spectra of CMCS hydrogel; (**f**) flowing solution state of Fe–Zr@PDA@CMCS hydrogel; (**g**) solidified state of Fe–Zr@PDA@CMCS hydrogel; (**h**) SEM image of Fe–Zr@PDA@CMCS hydrogel; (**i**) elemental distribution of Zr in Fe–Zr@PDA@CMCS hydrogel.

To examine the microstructural morphology, the nanoparticle was investigated with a scanning electron microscope (SEM, Zeiss Sigma 300, Oberkochen, Germany). Fe–Zr@PDA was synthesized by a hydrothermal one-pot method. The Fe–Zr@PDA NPs exhibited a homogeneous spherical morphology with a particle size of approximately 42.4 ± 9.2 nm (Figure 1a). The hydrodynamic diameters of NPs dispersed in different media were measured using DLS. As shown in Figure 1b, the mean hydrodynamic diameters of Fe–Zr@PDA in water was 164 nm. The results of DLS also revealed that Fe–Zr@PDA tended to undergo certain aggregation. More importantly, the hydrodynamic diameters of Fe–Zr@PDA hardly changed within 24 h. To verify the existence of iron, Fe–Zr@PDA was mixed with the o-phenanthroline solution at room temperature. It was observed that the supernatant changed swiftly from colorless to orange–red and showed the characteristic absorption at 510 nm. However, there was no detectable color change when cultured without Fe–Zr@PDA (Figure 1c). These results suggest the presence of Fe in the composite. Fe–Zr@PDA@CMCS hydrogel was prepared by mixing the Fe–Zr@PDA with the EDC/NHS-activated CMCS. SEM analysis showed that the CMCS hydrogel showed a smooth surface (Figure 1d). Compared to the unactivated CMCS [48], a new peak at 1650 cm^−1^ assigned to the -C=N was observed in the FT-IR spectrum of CMCS hydrogel (Figure 1e). These results suggest that CMCS forms amide bonds through the activation of EDC/NHS, which facilitated the formation of hydrogels. A digital camera was used to record the changes of the black solution (Figure 1f, g), indicating the solution state and solid state of the hydrogel. The macroscopic and microscopic appearance of hydrogel was then recorded, which uncovered that the hydrogel is large and in macro scale. PDA is capable of generating thin film coatings on the surface of multiple materials. As shown in Figure 1d, h, CMCS hydrogel showed a smooth surface, while the Fe–Zr@PDA@CMCS hydrogel showed some loose porous pores. This may be attributed to the formation of hydrogen bonds between PDA and CMCS. The element distribution diagram clearly showed that Zr were evenly distributed in the hydrogel, and there were no excess impurity elements in the hydrogel (Figure 1i).

### 2.2. Photothermal Conversion Evaluation of Fe–Zr@PDA@CMCS Hydrogel

**Figure 2:**
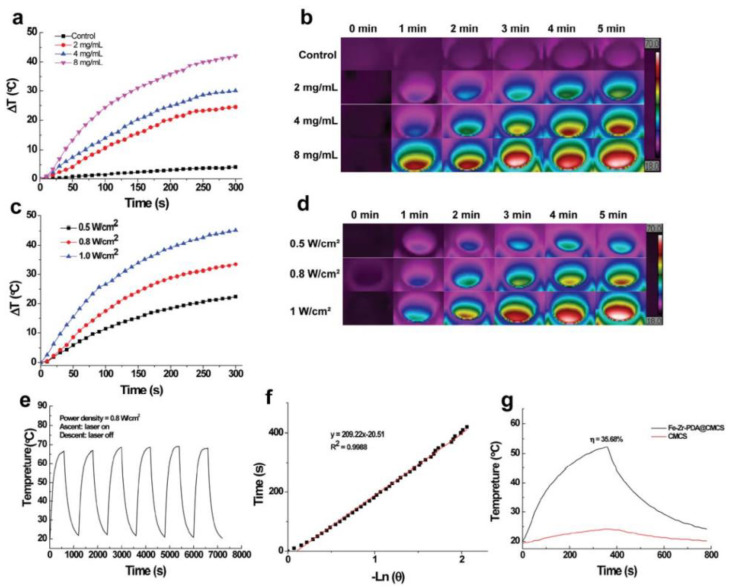
(**a**) Temperature variation of Fe–Zr@PDA@CMCS hydrogel with different Fe–Zr@PDA doping levels under 808 nm laser irradiation; (**b**) infrared thermographs correspond to (**a**), colors indicate different temperatures; (**c**) the temperature profiles of Fe–Zr@PDA@CMCS hydrogel irradiated with NIR laser with different power densities of 0.5, 0.8, and 1.0 W/cm^2^; (**d**) infrared thermographs correspond to (**c**), colors indicate different temperatures; (**e**) results from photothermal cycling tests results of Fe–Zr@PDA@CMCS hydrogel after 8 irradiation and cooling cycles; (**f**) time constant curves for Fe–Zr@PDA@CMCS hydrogel (808 nm, 1.0 W/cm^2^); (**g**) in vitro η values for Fe–Zr@PDA@CMCS hydrogel.

In recent years, great efforts have been made in developing photothermal agents for the therapy of various types of tumors [28]. PDA has good photothermal properties, can enhance the biocompatibility of nanomaterials, improve hydrophilicity, reduce cytotoxicity, and its many other advantages are widely used in the field of nanomaterials research [49,50]. The doping of PDA gives Fe–Zr@PDA@CMCS hydrogel the photothermal conversion properties to convert the absorbed NIR laser mild into heat for action. In this study, the temperatures of the hydrogel were recorded for the different groups of treatments to determine the overall photothermal performance of the hydrogels (Figure 2a–d). As shown in Figure 2a, as the concentration of Fe–Zr@PDA in the hydrogel increased (from 2 mg/mL to 8 mg/mL), and the ΔT gradually increased, with ΔT of 24.5 °C, 30 °C, and 42.1 °C, respectively, while the temperature of the control group only increased by 4.2 °C. The corresponding thermographs, likewise, illustrated that the concentration of PDA is closely related to the photothermal effect (Figure 2b). Further, the hydrogel was irradiated with an 808 nm NIR laser of exceptional energy densities (the specific power densities of the laser are 0.5 W/cm^2^, 0.8 W/cm^2^, and 1.0 W/cm^2^) for 5 min and the ΔT of the hydrogel was measured. The results revealed that the higher the power density of the NIR laser was, the higher the ΔT was (Figure 2c). This suggests that the power density of the NIR laser is another factor affecting its photothermal performance. The thermographic images provided further evidence of the power-density-related photothermal performance (Figure 2d). In addition, Fe–Zr@PDA@CMCS hydrogel has good photothermal stability. After being irradiated by the laser for up to 120 min (six NIR irradiated cycles), the hydrogel showed a stable temperature rise trend, and the maximum ΔT was still greater than 50 °C (Figure 2e). Based on linear regression analysis, we calculated that the τs of Fe–Zr@PDA@CMCS hydrogel was 209.22 s (Figure 2f), and the η was 35.68% (Figure 2g), which was higher than the Au@Bi_2_ Se_3_ core–shell nanoparticle [42].

### 2.3. •OH Generating Capacity of Fe–Zr@PDA@CMCS Hydrogel

**Figure 3:**
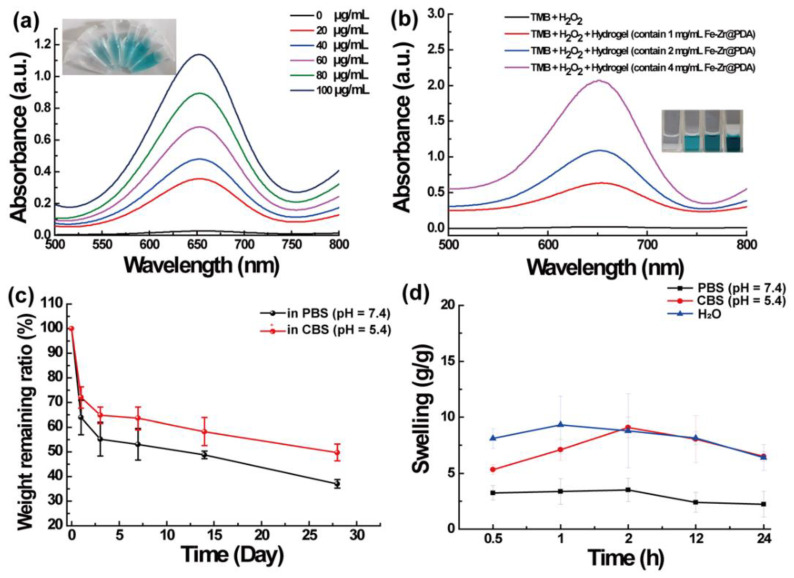
(**a**) Experimental results and corresponding digital camera photos of the reaction of different concentrations of Fe–Zr@PDA nanoenzymes with TMB and H_2_O_2_ to produce •OH; (**b**) absorbance values of different concentrations of Fe–Zr@PDA@CMCS after co-incubation with TMB and H_2_O_2_, and digital photographs of the supernatant photographic images; (**c**) degradation rates of Fe–Zr@PDA@CMCS hydrogel in CBS and PBS solutions; (**d**) swelling rate of Fe–Zr@PDA@CMCS hydrogel in ultrapure water, CBS solution, and PBS solution.

Therapeutic modalities based on reactive oxygen radicals and their derivatives have been demonstrated as emerging therapeutic strategies for tumors [43,51,52]. Among these, Fenton-based and Fenton-like responses are considered to be potential tumor cell therapy modalities and there are now several studies demonstrating the use of the Fenton effect to cause tumor-killing effects [44,45,46,47,53]. We first verified the ability of Fe–Zr@PDA to produce •OH; specifically, •OH can rapidly oxidize colorless TMB to a blue–green oxidized TMB compound (oxTMB), and further scanned the absorbance of the reacted solution at 652 nm by UV spectrophotometry. The magnitude of the absorbance gave a side view of the ability of the Fenton reaction to produce •OH. As shown in Figure 3a, the concentration of Fe–Zr@PDA was increased from 0 μg/mL to 100 μg/mL, and the results of the absorbance of the final solution after reaction with TMB were gradually increased, indicating a positive correlation between its ability to produce •OH and the concentration of Fe–Zr@PDA. The reaction of Fe–Zr@PDA with H_2_O_2_ shows that the colorless TMB is oxidized to the blue oxTMB, which further demonstrates the ability of Fe–Zr@PDA to generate •OH (Figure 3a). Further, we tested the •OH generation ability of Fe–Zr@PDA@CMCS hydrogel. The control hydrogel without Fe–Zr@PDA@CMCS had an absorbance value of almost zero at 652 nm and the mixture remained transparent (Figure 3b). In contrast, the absorbance of Fe–Zr@PDA@CMCS hydrogel increased with the concentration of Fe–Zr@PDA and the color of the solution deepened, which further indicated that the catalytic ability of Fe–Zr@PDA was well-preserved after doping in the hydrogel (Figure 3b).

### 2.4. Evaluation of Hydrogel Degradation and Swelling Properties

The ability to swell and degrade swelling properties are important physical parameters of hydrogels, which affect the rate of release of loaded compounds and the absorption of tissue exudate, among other things [54,55,56]. In this section, we first investigated the degradation properties of Fe–Zr@PDA@CMCS hydrogel under different pH conditions. As shown in Figure 3c, Fe–Zr @PDA@CMCS hydrogel showed a fast weight loss in both PBS and CBS at the beginning and then degraded by more than 45% in the first three days. The degradation rate increased gradually with time, but the degradation rate was slower in the CBS than in the PBS. It is worth mentioning that the TME is acidic due to the altered metabolism of the tumor cells; therefore, the Fe–Zr@PDA@CMCS hydrogel can be retained in the acidic TME for a longer period to achieve prolonged drug release and tumor therapy.

Then, we evaluated the swelling properties of Fe–Zr@PDA@CMCS hydrogel by immersing them in ultrapure water, CBS, or PBS for 24 h. As shown in Figure 3d, the freeze-dried hydrogel immersed in PBS buffer and ultrapure water swelled hastily inside a brief length when they come upon the liquid. However, the swelling state of the hydrogel immersed in the PBS buffer solution also increased slowly with time. At the same time, the swelling of the hydrogel in pure water remained almost constant, and in the final experimental phase, the freeze-dried hydrogel reached swelling equilibrium. Interestingly, compared with the hydrogel soaked in PBS and pure water, Fe–Zr@PDA@CMCS hydrogel soaked in CBS showed little change in the swelling degrees. This will facilitate the presence of Fe–Zr@PDA@CMCS hydrogel in a slightly acidic environment.

### 2.5. In Vitro Cytocompatibility Results of Fe–Zr@PDA@CMCS Hydrogel

**Figure 4:**
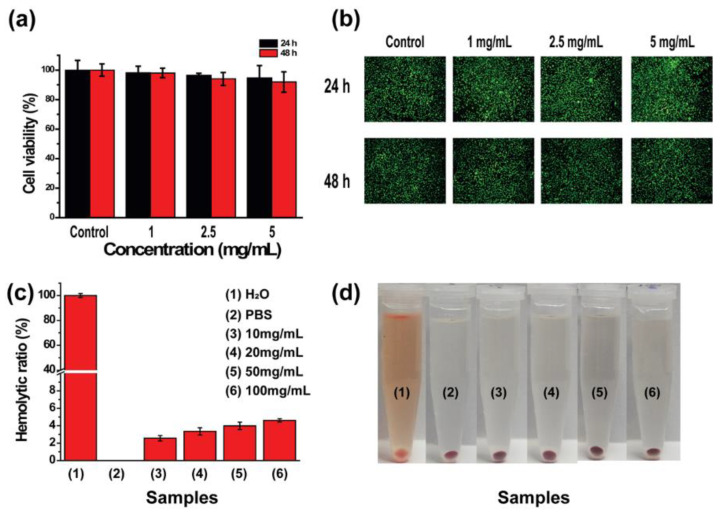
(**a**) Cell viability of Fe–Zr@PDA@CMCS hydrogel extracts at 1 mg/mL, 2.5 mg/mL, and 5 mg/mL co-cultured with mouse fibroblasts (L929 cell line) for 24 h and 48 h; (**b**) AM-PI stained images of L929 cell lines co-cultured with Fe–Zr@PDA@CMCS hydrogel extracts at 1 mg/mL, 2.5 mg/mL, and 5 mg/mL for 24 h and 48 h; (**c**) hemolysis ratio of rat erythrocytes after co-culture with different concentrations of Fe–Zr@PDA@CMCS hydrogel; (**d**) photos of rat erythrocytes after centrifugation.

Biocompatibility refers to the ability of a material to elicit appropriate host and material responses in a given application environment [57,58]. The ideal composite hydrogel requires good biocompatibility both in vitro and in vivo [59,60]. We evaluated the cell safety of Fe–Zr@PDA@CMCS hydrogel through a series of in vitro cell experiments using the mouse fibroepithelial L929 cell line as a model. Adherently grown L929 cells were co-cultured with 1 mg/mL, 2.5 mg/mL, and 5 mg/mL of hydrogel extracts for 24 h and 48 h, and the results are shown in Figure 4a. Even at concentrations up to 5 mg/mL, the viability of L929 cells at 24 h and 48 h was higher than 90%, indicating that Fe–Zr@PDA@CMCS hydrogel has good cytocompatibility (Figure 4a). Following the CCK-8 cell viability assay, we further stained cells in each experimental and control group using AM-PI stain and observed the distribution of live and dead cells using inverted phase contrast fluorescence microscopy. As can be seen in Figure 4b, in the 24 h and 48 h images, both in the control and experimental groups, the l929 cells, which represent viable cells, show green fluorescence, while the red fluorescence, which represents dead cells, is negligible in the images. It is worth noting that there is no significant difference in the observation of cell morphology between the experimental group and the control group. The above staining results were consistent with the results of CCK-8 experiments, which further demonstrated that Fe–Zr@PDA@CMCS hydrogel has no significant effect on the cell morphology of L929 cells and has good cell safety.

### 2.6. In Vitro Hemocompatibility Results of Fe–Zr@PDA@CMCS Hydrogel

The validation of in vitro biocompatibility experiments is crucial to ensure the biomedical applications of the Fe–Zr@PDA@CMCS hydrogel. The hemocompatibility test was used to assess whether Fe–Zr@PDA@CMCS hydrogel would cause hemolysis. Briefly, Fe–Zr@PDA@CMCS hydrogel was co-cultured with rat red blood cells to investigate their hemocompatibility. As shown in Figure 4c, the hemolysis rate of erythrocytes was 2.56 ± 3.31%, 3.35 ± 0.41%, 3.98 ± 0.43%, and 4.61 ± 0.18% after being cultured with Fe–Zr@PDA@CMCS hydrogel with concentrations of 10, 20, 50, and 100 mg/mL respectively, which were all less than 5%. This result indicated that Fe–Zr@PDA@CMCS hydrogel has good hemocompatibility. In addition, we recorded digital photographs of the erythrocyte solution after co-culture and, in agreement with the previous results, the positive control group of erythrocytes co-cultured with ultrapure water appeared bright red. This demonstrated that the erythrocytes have all ruptured, whereas the supernatant of the experimental group was clearer and more transparent, indicating that most of the erythrocyte structure was still intact (Figure 4d).

### 2.7. In Vitro Biocompatibility Assessment Results of Fe–Zr@PDA@CMCS Hydrogel

**Figure 5:**
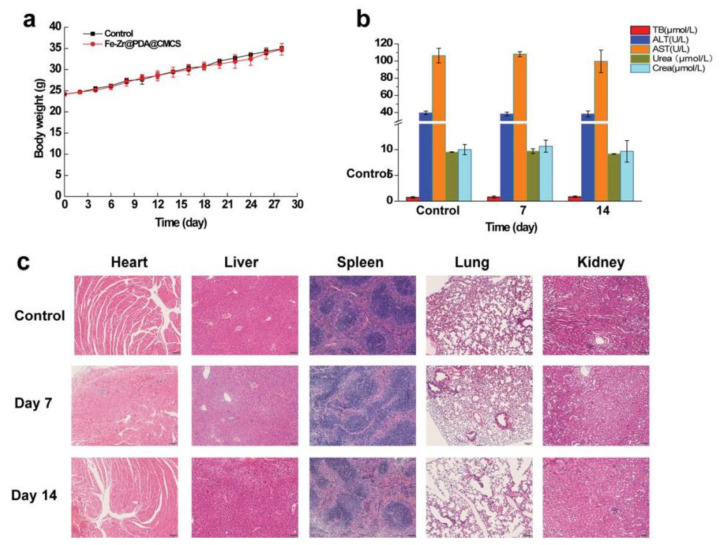
(**a**) Changes in body weight of Fe–Zr@PDA@CMCS hydrogel treated mice and normal mice (control) at day 28; (**b**) results of serum biochemical parameters of Fe–Zr@PDA@CMCS hydrogel treated mice and normal mice (control); (**c**) H&E staining of the main organs of the Fe–Zr@PDA@CMCS hydrogel treated mice and normal mice (control). Bar = 200 μm.

**Figure 6:**
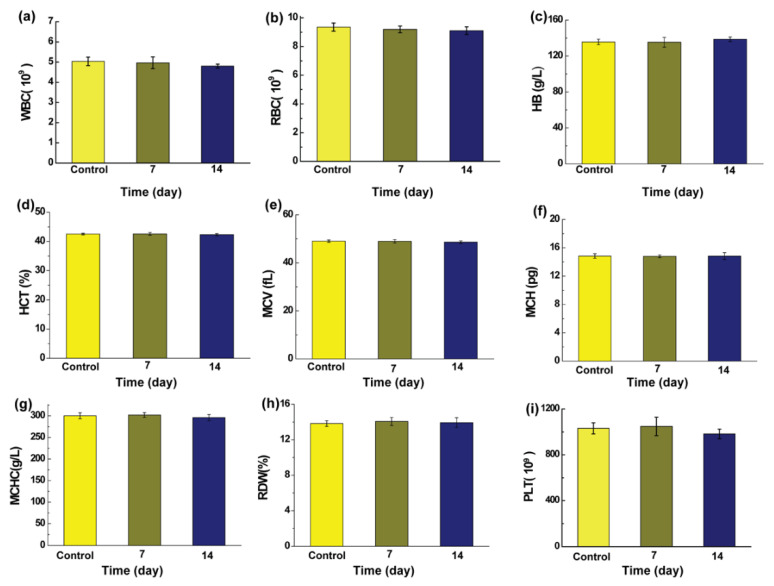
Routine blood test data of Fe–Zr@PDA@CMCS hydrogel-treated mice and normal mice (control): (**a**) white blood cell (WBC), (**b**) red blood cell (RBC), (**c**) hemoglobin (HB), (**d**) red blood cell-specific volume (HCT), (**e**) mean corpuscular volume (MCV), (**f**) mean corpuscular hemoglobin (MCH), (**g**) mean corpuscular hemoglobin concentration (MCHC), (**h**) red cell volume distribution width (RDW), and (**i**) PLATELET (PLT).

After establishing the excellent in vitro cytocompatibility of Fe–Zr@PDA@CMCS hydrogel, the safety of the hydrogel was further assessed in vivo by subcutaneously embedding Fe–Zr@PDA@CMCS hydrogel in KM mice and recording the changes in body weight of the mice. The body weight of mice in both the experimental and control groups increased normally throughout the feeding cycle, and the body weight of mice in the experimental group fluctuated slightly for 21 days after encapsulation in the hydrogel; however, there was no statistical difference compared to the control group (*p* > 0.05) (Figure 5a). Further, we measured the blood biochemical parameters of the experimental and control mice on day 7 and 14 of the hydrogel implantation to assess whether the liver and kidney functions were affected. The serum biochemical parameters measured included TB (total bilirubin), ALT (alanine aminotransferase), AST (aspartate aminotransferase), UREA (urea), and CREA (creatinine). As shown in Figure 5b, the experimental and control groups of the hydrogel did not show a statistically significant difference in liver and kidney function (*p* > 0.05), which could further confirm the safety of the hydrogel. Next, we evaluated H&E staining sections of the major organs after 7 and 14 days of feeding, which showed no significant cellular hypertrophy, atrophy, or necrosis in any of the organ tissue sections. These results further demonstrated that the encapsulated Fe–Zr@PDA@CMCS hydrogel had no significant side effects on the normal physiological function of the major organs and had a good in vivo safety profile (Figure 5c). Finally, we examined the routine blood parameters of each group of mice, and the results are shown in Figure 6a–i. There was no statistical difference (*p* > 0.05) between the experimental and control groups of mice at 7 and 14 days.

### 2.8. In Vitro Anticancer Potential of Fe–Zr@PDA@CMCS Hydrogel

**Figure 7:**
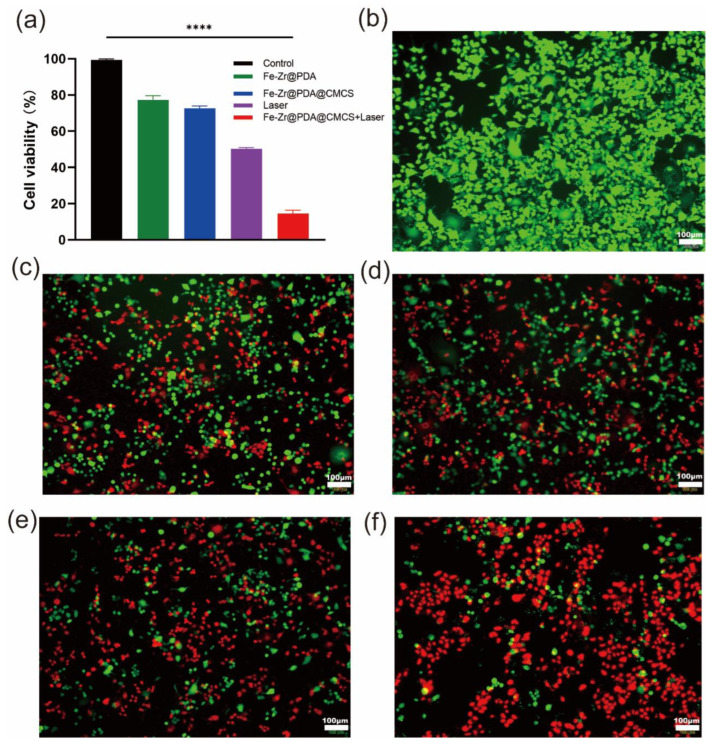
(**a**) CCK-8 results of cells after different treatments; (**b**–**f**) results of live and dead cell staining of SW1990 cells, morphological staining images of cells corresponding to CCK-8 results (**b**): L929 cells co-incubated with fresh medium; (**c**): L929 cells co-incubated with 100 μg/mL Fe–Zr@PDA nanoparticles; (**d**): L929 cells co-incubated with 100 μg/mL Fe–Zr@PDA@CMCS hydrogel extract; (**e**): L929 cells irradiated by 808 nm NIR, 1 W/cm^2^, 5 min pure photothermal irradiation; (**f**): fluorescent staining images of L929 cells after treatment with 100 μg/ mL Fe–Zr@PDA@CMCS hydrogel extract and 808 nm NIR, 1 W/cm^2^, by 5 min of light. Bar = 100 μm, **** *p* < 0.0001.

To investigate their potential effectiveness in antitumor therapy, we selected SW1990 cells for in vitro evaluation of tumor therapy. The CCK-8 results (Figure 7a) showed that there was a decrease in the survival rate of SW1990 cells in the Fe–Zr@PDA nanoparticles group, proving that Fe–Zr@PDA caused some damage to the normal growth of cancer cells. The Fe–Zr@PDA@CMCS hydrogel extract group also showed a decrease in cell survival rate, suggesting that CMCS-doped functionalized hydrogels also have some tumor-cell-killing effect. In contrast, the survival rate of SW1990 cells receiving photothermal treatment alone was significantly lower, with almost half of the cells being inactive. Most importantly, the Fe–Zr@PDA@CMCS hydrogel extract combined with photothermal treatment showed an even more pronounced reduction in cell survival, indicating that the synergistic Fe–Zr@PDA@CMCS hydrogel and photothermal treatment can kill even more tumor cells. Afterwards, we further validated the in vitro anticancer effects by live–dead cell staining. The red fluorescence of Fe–Zr@PDA nanoparticles, Fe–Zr@PDA@CMCS hydrogel extracts treated group and photothermal treatment group all showed a significant increase compared to the control group (Figure 7b–e). Notably, there was a significant increase in red fluorescence and a significant decrease in green fluorescence after combining Fe–Zr@PDA@CMCS hydrogel with photothermal treatment, indicating that most of the SW1990 tumor cells were killed (Figure 7f). In conclusion, the combination of multifunctional hydrogel and photothermal therapy can effectively kill tumor cells, which is also expected to further enable tumor treatment at the in vivo level again.

## 3. Conclusions

In summary, we successfully designed and prepared multifunctional Fe–Zr@PDA@CMCS hydrogel with the Fenton effect and photothermal conversion properties using Fe, Zr, PDA, and CMCS. Fe–Zr@PDA@CMCS hydrogel combined the advantages of hydrogel and nanoparticles. The photothermal conversion, degradation, and swelling capabilities of the hydrogel and Fenton’s catalytic ability under different conditions were investigated. The results showed that the composite hydrogel retains its photothermal and Fentonian catalytic properties while protecting Fe–Zr@PDA nanoparticles from degradation. In addition, the good biocompatibility of the hydrogel was demonstrated at the cellular and animal levels. Further results demonstrated good therapeutic effects at the cellular level, and will be validated at the animal level in the future. Unfortunately, there are limitations to our study and we need further validation at the animal level and clinical level. In summary, multifunctional composite hydrogels can be used as carriers of drugs for multimodal tumor therapy and tissue regeneration for biomedical applications. Based on the good degradability and therapeutic effects of functionalized hydrogels, multifunctional composite hydrogels could provide novel ideas and show great promise for future clinical oncology treatments. The study is also expected to provide a reference for future clinical studies of novel hydrogels against tumor recurrence.

## 4. Materials and Methods

### 4.1. Materials

All chemicals were used without further purification. Zirconium tetrachloride (ZrCl_4_), ferric chloride hexahydrate (FeCl_3_-6H_2_O), CMCS, PDA, ethylene glycol (EG), EDC, and NHS were purchased from Shanghai Aladdin Reagent Co., Ltd. (Shanghai, China). Mouse fibroblast cells (L929) and human pancreatic cancer cell lines (SW1990) were bought from the Institute of Biochemistry and Cell Biology, Chinese Academy of Sciences (Shanghai, China). Dulbecco’s Modified Eagle Medium (DMEM), citrate buffer solution (CBS), and phosphate buffer saline (PBS) were procured from Gibco Co., Ltd. (Shanghai, China). The Cell counting kit-8 (CCK-8) was purchased from Vazyme Biotechnology Co., Ltd. (Nanjing, China). Calcein-AM/PI Live/Dead kit was purchased from Solarbio Technology (Beijing, China) Co., Ltd. Kunming (KM) mice (female, 4–6 weeks, 20–25 g) and Sprague-Dawley (SD) rats were ordered from Shanghai Slac Laboratory Animal Center (Shanghai, China). All experimental mice were fed strictly following the rules and regulations of the Ministry of Health of the People’s Republic of China.

### 4.2. Preparation of Fe–Zr@PDA

The Fe–Zr@PDA nanoparticles were synthesized by a simple one-pot hydrothermal method. Specifically, 0.2 g of FeCl_3_–6H_2_O and 0.075 g of ZrCl_4_ were first weighed in scales and dissolved in 15 mL of ethylene glycol with stirring at room temperature, and the dissolved solution was named solution A. Next, 0.492 g of sodium acetate and 0.2 g of dopamine were dissolved in 10 mL of ethylene glycol (to form solution B). Solution A and solution B were then mixed well and added to a 100 mL autoclave lined with polytetrafluoroethylene, which was placed in an oven set at 180 °C for 12 h. Finally, after the reaction had cooled naturally, the product was washed 3 times with deionized water and anhydrous ethanol, centrifuged (9000 rpm, 8 min) to obtain the desired product, freeze-dried in a lyophilizer, and stored for subsequent experiments.

### 4.3. Synthesis of CMCS Hydrogel and Fe–Zr@PDA@CMCS Hydrogel

To synthesize the CMCS hydrogel, as a first step, 0.1 g of CMCS was introduced to 2 mL of ultrapure water, then dissolved while stirring in a water bath set to 50 °C. Next, 0.1 g of the activator EDC (0.05 g)/NHS (0.05 g) was added to 1 mL of pure water and the solution containing the activator was added to the CMCS solution and further stirred to form a gel. To synthesize the Fe–Zr@PDA@CMCS hydrogel, in brief, 0.05 g of EDC and 0.05 g of NHS were delivered to a solution of Fe–Zr@PDA nanomaterials (1 mL with a concentration of 0.01 g/mL) and finally mixed with the CMCS solution and stirred to form the hydrogel.

### 4.4. Characterizations of Fe–Zr@PDA@CMCS and Fe–Zr@PDA

To observe the microscopic morphology and elemental distribution of the CMCS and Fe–Zr@PDA@CMCS hydrogel, firstly, the conductive glue was carefully pasted on the test sample table, and the freeze-dried CMCS and Fe–Zr@PDA@CMCS hydrogel were cut into thin pieces and placed on the conductive glue. Then, an infrared baking lamp was used to dry the surface of the hydrogel. After that, its morphology was examined using a scanning electron microscope (SEM, Zeiss Sigma 300). Similarly, the morphology and particle size of Fe–Zr@PDA were observed using SEM, and the elemental composition of Fe–Zr@PDA nanoparticles was further analyzed using an X-ray energy spectrometer.

### 4.5. Characteristics of Photothermal Conversion

To explore the influence of different power densities on the temperature change, Fe–Zr@PDA@CMCS hydrogel was irradiated with the near-infrared laser electricity with strength densities of 0.5 W/cm^2^, 0.8 W/cm^2^, or 1 W/cm^2^. Then, we recorded the temperature change using an FLIR^E60^ camera. Finally, the photothermal stability of the hydrogel was explored. Specifically, the near-infrared laser with a power density of 0.8 W/cm^2^ was used to irradiate the hydrogel for 6 cycles, and the cycle was set at 20 min each time. During the experiment, the thermal images and temperature changes of the experimental each cycle were recorded using a near-infrared thermal imager. The photothermal conversion efficiency (η) of Fe–Zr@PDA@CMCS was calculated by the modified Korgel formula (1):

(1) $$\eta \left(\%\right)=\frac{hS\left(T_{max}-T_{surr}\right)-Q_{in.surr}}{I\left(1-10^{\left(-{A(}_{\lambda })\right)}\right)}$$

In the above formula, η is the photothermal conversion efficiency, *h* is the warmness switch coefficient, *I* replace the power of the laser, *T_max_* is the highest temperature of Fe–Zr@PDA@CMCS solution under the laser irradiation for 6 min, *T_surr_* represents the ambient temperature throughout the test period, *hS* is the heat transfer coefficient, *A*(*_λ_*) is the absorbance of Fe–Zr@PDA@CMCS at wavelength 808 nm, *S* is the effective area of laser radiation, and *Q_in.surr_* is the heat loss of Fe–Zr@PDA@CMCS in the heating process.

### 4.6. •OH Generating Capacity

Firstly, we explored the ability of different concentrations of Fe–Zr@PDA to produce •OH. Specifically, a 3,3′,5,5′-Tetramethylbenzidine (TMB) solution with a concentration of 3.2 mM was prepared using ultrapure water and 300 μL was mixed with different concentrations of Fe–Zr@PDA and H_2_O_2_. The total volume of the mixed solution was 1.5 mL, with the final concentration of H_2_O_2_ set at 8 mM and the final concentrations of Fe–Zr@PDA set at 0 μg/mL, 20 μg/mL, 40 μg/mL, 60 μg/mL, 80 μg/mL, and 100 μg/mL. The reaction lasted for 15 min at room temperature accompanied by ultra-high-speed centrifugation (15,000 rpm, 10 min). After the reaction, the supernatant was collected and photographed with a digital camera. At the same time, the absorbance of each sample supernatant at the wavelength of 652 nm was measured by an ultraviolet spectrometer.

The •OH formation ability of Fe–Zr@PDA@CMCS hydrogel was also determined by the color development properties of TMB after oxidation. Fe–Zr@PDA@CMCS hydrogel with different concentrations (1, 2, or 4 mg/mL) was mixed with TMB (mM) and H_2_O_2_ (0.3%). The group of TMB and H_2_O_2_ without Fe–Zr@PDA@CMCS hydrogel was set as the control. After being co-cultured at room temperature for 1 h, the supernatant was accumulated and photographed with a digital camera. The absorption value of the supernatant at λ = 652 nm was measured by UV–Vis–NIR spectroscopy (Lambda 25, Perkin Elmer, Waltham, MA, USA).

### 4.7. Degradation Analysis of Fe–Zr@PDA@CMCS Hydrogel

To explore the in vitro degradation ability, lyophilized Fe–Zr@PDA@CMCS hydrogel (100 mg) was immersed in 5 mL of PBS (pH = 7.4) or CBS (pH = 5.4). Then, the Fe–Zr@PDA@CMCS hydrogel samples were removed at predetermined time points (1, 3, 7, 14, and 28 days), washed with ultrapure water, and finally lyophilized and weighed in a lyophilizer. The degradation percentage of Fe–Zr@PDA@CMCS hydrogel was once calculated by way of the following formula:

(2) $$Weight\;remaining\;ratio\left(\%\right)=\frac{R_{t}}{R_{0}}\times 100\%$$

where *R_t_* represents the actual gram weight of Fe–Zr@PDA@CMCS hydrogel and *R*_0_ represents the original weight of Fe–Zr@PDA@CMCS hydrogel.

### 4.8. Swelling Analysis of Hydrogel

The swelling rate (SR) and stability of Fe–Zr@PDA@CMCS hydrogel in different solutions (H_2_O, PBS, and CBS) were determined by the swelling test. In short, 10 mg of Fe–Zr@PDA@CMCS hydrogel was placed into a sealed centrifuge tube (*n* = 3) and added to 20 mL of H_2_O, PBS, and CBS respectively. The tubes were placed at 37 °C. The hydrogel was removed from the solution at predetermined time points (0.5, 1, 2, 12, and 24 h), and the surface water of the hydrogel was absorbed by filter paper and weighed again. The swelling kinetics curve of Fe–Zr@PDA@CMCS hydrogel was drawn, and the SR of the hydrogel reached swelling equilibrium was calculated according to the following formula:

(3) $$Swelling\;ratio\;(\%)=\frac{m_{t}-m_{0}}{m_{0}}\times 100\%$$

where *m_t_* is the mass of hydrogel after swelling at different points in time and *m*_0_ represents the initial condition of this hydrogel.

### 4.9. In Vitro Cytocompatibility of Fe–Zr@PDA@CMCS Hydrogel

The cell safety of Fe–Zr@PDA@CMCS was evaluated using mouse fibroblasts (L929 cells). First, the L929 cells were inoculated on 96-well clear cell culture plates (5000 cells per well) and cultured for 24 h in a constant temperature incubator with 37 °C and 5% CO_2_. At the same time, Fe–Zr@PDA@CMCS hydrogel was positioned in DMEM cell medium (1, 2.5, or 5 mg hydrogel, in 1 mL DMEM medium) and incubated at 37 °C in a single day to attain the extract with the attention of 5 mg/mL. On the second day, the old medium was replaced with 100 μL of the leaching solution, and the control group was added with a fresh DMEM medium. The extract concentration in the leaching solution was 1 mg/mL, 2.5 mg/mL, or 5 mg/mL. Finally, the medium was removed at 24 h or 48 h culture, and the cells were washed with PBS twice. Cell viability was determined by the CCK-8 kit. In addition, staining was performed using an AM-PI staining kit for both living and dead cells, and staining images were collected using an inverted phase contrast microscope (Leica DM IL, Weztlar, Germany).

### 4.10. In Vitro Blood Compatibility of Fe–Zr@PDA@CMCS Hydrogel

The feeding and testing of animals were conducted at Changhai Hospital of Naval Medical University in strict accordance with the program and policies of the Ministry of Health. To test whether Fe–Zr@PDA@CMCS can cause rupture of red blood cells and hemolysis, the blood compatibility of these cells was evaluated by using rat red blood cells. Firstly, blood was collected from the anesthetized heart of SD rats into anticoagulant collection vessels and centrifuged (4000 rpm, 5 min) to collect the red blood cells. Finally, the purified rat red blood cells were diluted to 2% with PBS and stored in the refrigerator at 4 °C for subsequent use. For the hemolysis experiment, we first mixed previously stored diluted erythrocyte suspensions with PBS buffer containing 20, 40, 100, and 200 mg of Fe–Zr@PDA@CMCS hydrogel, respectively. In addition, a negative control group (specifically, 1 mL of diluted PBS buffer mixed with 1 mL blood cells) and a positive control group (that is, 1 mL of diluted red cell suspension mixed with 1 mL ultrapure water) were set up, respectively. Finally, the above-mixed solutions were incubated in a water bath at 37 °C for 2 h and then centrifuged immediately to obtain the supernatant (3000 rpm, 5 min). The absorbance at 541 nm was measured with the UV–visible–near-infrared spectrometer (Lambda 25, Perkin Elmer, Waltham, MA, USA) of all the supernatants collected. Then, the hemolysis percentage (HP) was calculated according to the following formula, and the supernatant was photographed with a camera.

(4) $$Hemolytic\;ratio\left(\%\right)=\frac{B_{sample}-B_{negative}}{B_{positive}-B_{negative}}\times 100\%$$

where *B_sample_* is the absorbance at 541 nm of red blood cell suspension after treatment with hydrogel extract, *B_negative_* is the absorbance of erythrocyte suspension dealt with PBS buffer, and *B_positive_* is the absorbance of blood cell suspension after treatment with ultrapure water.

### 4.11. In Vivo Animal Tissue Safety of Fe–Zr@PDA@CMCS Hydrogel

To explore the safety of Fe–Zr@PDA@CMCS hydrogel in animals, KM mice were used as the animal model. Fe–Zr@PDA@CMCS hydrogel was embedded under the skin of mice to observe whether it would cause damage to mice. Specifically, KM mice (from the Laboratory in Shanghai Changhai Hospital, China) were randomly divided into control and experimental groups with three animals in each group (*n* = 3). Mice anesthetized with pentobarbital sodium were subcutaneously implanted with 200 mg of Fe–Zr@PDA@CMCS hydrogel in the experimental group. Normal healthful mice were used in the control group. The mice were weighed every 2 days after embedding to investigate the adjustments in body weight. The mice were sacrificed on the 7th, 14th, and 28th days, respectively. We collected blood by removing the eyes of mice, and major organs such as lungs, spleen, liver, heart, and kidney were collected and fixed in 4% paraformaldehyde for HE staining. Meanwhile, the collected blood was used to measure the values of the blood routine indexes of the two groups by the blood routine analyzer. An ELISA kit was used to detect the related indexes of kidney function and liver function in the serum. HE-stained sections of major organs were used to observe whether there were lesions such as inflammation and necrosis. All animal experimental operations were carried out in strict accordance with the protocols authorized by the hospital’s Comprehensive Laboratory Animal Centre.

### 4.12. In Vitro Antitumor Effects of Fe–Zr@PDA@CMCS Hydrogels

We selected the human pancreatic cancer cell line SW1990 as a model to assess the in vitro therapeutic effect of Fe–Zr@PDA@CMCS. SW1990 cells were firstly inoculated into the 96-well culture plate at a density of 9000 cells/well. After 24 h of incubation, the original medium was replaced with 100 μL of fresh DMEM solution containing different substances and subjected to various treatments. The treatments were as follows: (a) control group (fresh DMEM), (b) Fe–Zr@PDA group (100 μg/mL Fe–Zr@PDA), (c) Fe–Zr@PDA@CMCS group (100 μg/mL Fe–Zr@PDA hydrogel extract), (d) laser group (808 nm NIR, 1 W/cm^2^, 5 min), (e) Fe–Zr@PDA@CMCS + laser group (100 μg/mL Fe–Zr@PDA hydrogel extract, 808 nm NIR, 1 W/cm^2^, 5 min). The groups were treated as described above and incubation was continued for 12 h, after which the survival of SW1990 cells was assessed by the CCK-8 method and the live–dead cell assay.

### 4.13. Statistical Analysis

All results are expressed as mean ± standard deviation and one-way ANOVA statistical analysis was used to assess the significance of the experimental data. The value of 0.05 was used as the significance level data, with probabilities less than 0.05 (*p* < 0.05), 0.01 (*p* < 0.01), and 0.001 (*p* < 0.01) indicated by (*), (**), and (***), respectively. The sample size was 3 unless stated (*n* = 3).

## Data Availability

The data that support the findings of this study are available from the corresponding author upon reasonable request.
